# MRI-based quantification of posterior ocular globe flattening during 60 days of strict 6° head-down tilt bed rest with and without daily centrifugation

**DOI:** 10.1152/japplphysiol.00082.2022

**Published:** 2022-11-03

**Authors:** Stuart H. Sater, Gabryel Conley Natividad, Akari J. Seiner, Audrey Q. Fu, Dev Shrestha, Eric M. Bershad, Karina Marshall-Goebel, Steven S. Laurie, Brandon R. Macias, Bryn A. Martin

**Affiliations:** ^1^Alcyone Therapeutics Inc., Lowell, Massachusetts; ^2^Department of Chemical and Biological Engineering, University of Idaho, Moscow, Idaho; ^3^Department of Mathematics and Statistical Science, Institute of Bioinformatics and Evolutionary Studies, Institute for Modeling Collaboration & Innovation, University of Idaho, Moscow, Idaho; ^4^Department of Neurology, Baylor College of Medicine, Houston, Texas; ^5^KBR, Houston, Texas; ^6^Cardiovascular and Vision Laboratory, Johnson Space Center, National Aeronautics and Space Administration, Houston, Texas

**Keywords:** artificial gravity, head-down tilt bed rest, magnetic resonance imaging, ocular globe flattening, spaceflight associated neuro-ocular syndrome

## Abstract

Spaceflight associated neuro-ocular syndrome (SANS) is associated with acquired optic disc edema, hyperopia, and posterior globe flattening in some astronauts during long-duration spaceflight possibly due to the headward fluid redistribution in microgravity. The goal of this study was to assess whether strict head-down tilt (HDT) bed rest as a spaceflight analog would produce globe flattening and whether centrifugation could prevent these changes. Twenty-four healthy subjects separated into three groups underwent 60 days of strict 6° HDT bed rest: one control group with no countermeasure (*n* = 8) and two countermeasure groups exposed to 30 min daily of short-arm centrifugation as a means of artificial gravity (AG), either intermittent (iAG, *n* = 8) or continuous (cAG, *n* = 8). Magnetic resonance images (MRI) were collected at baseline, *HDT-day 14*, *HDT-day 52*, and 3 days after bed rest. An automated method was applied to quantify posterior globe volume displacement compared with baseline scans. On average, subjects showed an increasing degree of globe volume displacement with bed rest duration (means ± SE: 1.41 ± 1.01 mm^3^ on HDT14 and 4.04 ± 1.19 mm^3^ on HDT52) that persisted post-bed rest (5.51 ± 1.26 mm^3^). Application of 30 min daily AG did not have a significant impact on globe volume displacement (*P* = 0.42 for cAG and *P* = 0.93 for iAG compared with control). These results indicate that strict 6° HDT bed rest produced displacement of the posterior globe with a trend of increasing displacement with longer duration that was not prevented by daily 30 min exposure to AG.

**NEW & NOTEWORTHY** Head-down tilt (HDT) bed rest is commonly used as a spaceflight analog for investigating spaceflight associated neuro-ocular syndrome (SANS). Posterior ocular globe flattening has been identified in astronauts with SANS but until now has not been investigated during HDT bed rest. In this study, posterior ocular globe volume displacement was quantified before, during, and after HDT bed rest and countermeasures were tested for their potential to reduce the degree of globe flattening.

## Graphical abstract

**Figure:**
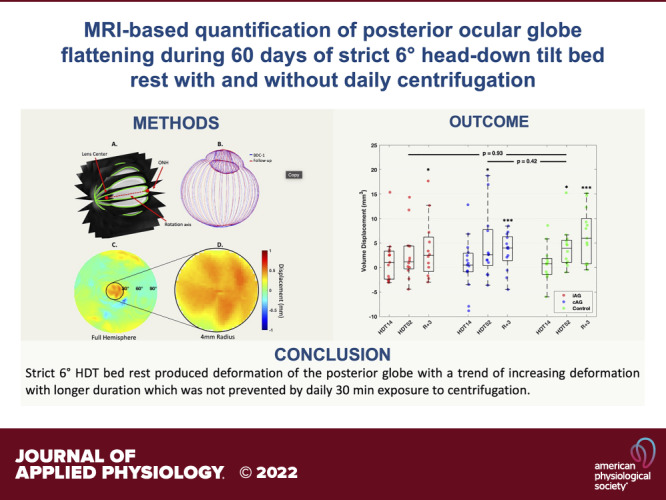

## INTRODUCTION

**Figure 1.**
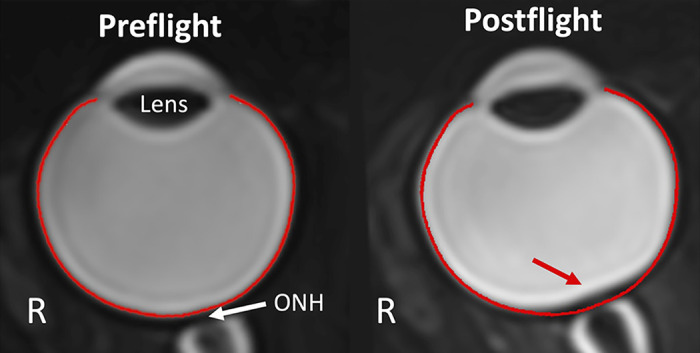
Posterior globe flattening near the optic nerve head (ONH) has been identified subjectively and objectively after long-duration spaceflight and as a major symptom of spaceflight associated neuro-ocular syndrome (SANS). A contour (red) is overlayed on a preflight and postflight T2-weighted MRI to show globe flattening that occurred after long-duration spaceflight that corresponds to a volume displacement of 39.2 mm^3^. This image was obtained as a part of the NASA Ocular Health Study (7).

Ophthalmic structural and visual acuity changes in a condition known as spaceflight associated neuro-ocular syndrome (SANS) have been observed in astronauts during and after long-duration spaceflight (>4 mo) (1–4). The proportion of astronauts who present with optic disc edema, a symptom of SANS, is ∼70% (5) but many of these cases are not clinically relevant and the proportion of astronauts who are diagnosed with SANS varies over time due to the ongoing characterization of its formal criteria (6). SANS findings include ocular changes such as choroidal folds, optic disc edema, hyperopic shift, and posterior globe flattening (Fig. 1) (6). In some subjects, these changes have been shown to persist long after return to Earth (7–9). Changes in cerebrospinal fluid (CSF) and hemodynamics that occur in microgravity in the absence of a gravity-induced hydrostatic pressure gradient are thought to be primary contributors to the development of SANS findings (10). In addition, long-term increases in intracranial blood and/or cerebrospinal fluid (CSF) pressure relative to seated or standing position (10) may result in a reduction of the translaminar pressure difference (TLPD) in the posterior globe/perioptic nerve region, wherein the difference between CSF pressure in the optic nerve sheath and intraocular pressure (IOP) is chronically lowered, contributing to scleral flattening and/or remodeling (11, 12).

Ground-based spaceflight analogs are utilized to study the simulated effects of microgravity including the sustained headward fluid shift. Currently, strict 6° head-down tilt (HDT) bed rest studies are used as a spaceflight analog for understanding some features of SANS, as the chronic HDT position simulates the headward fluid redistribution that occurs in microgravity and has resulted in the development of optic disc edema (13, 14) and the appearance of chorioretinal folds in the same cohort of subjects reported on here (15), albeit with preservation of the *Gx* (chest to back) vector. However, HDT studies may overestimate fluid redistributions compared with microgravity (14). This suggests that ophthalmic changes may be more sensitive to HDT duration than spaceflight duration, allowing for shorter study durations.

Many adaptations and physiological responses to spaceflight are attributed to the lack of a gravitational gradient during spaceflight. This has led researchers to hypothesize that simulating gravitational forces with centrifugal forces could mitigate the negative effects of microgravity by counteracting headward fluid shifts (16). In short-term HDT bed rest studies, short-arm centrifugation was an effective countermeasure for musculoskeletal deconditioning (17) and orthostatic tolerance (18) in subjects. Both studies noted that intermittent centrifugation was more effective than continuous centrifugation in reducing the effects of HDT, and daily centrifugation was well tolerated by participants (19).

**Figure 2.**
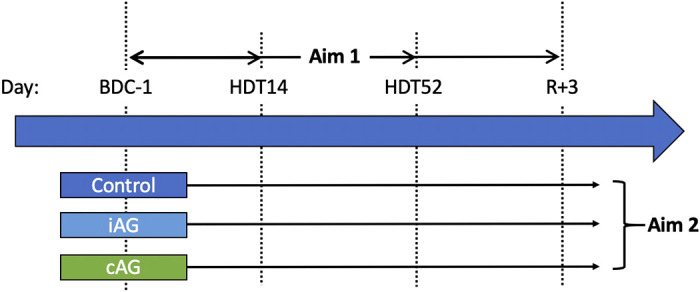
Schematic summarizing the study design and study aims. Subjects were split into three study groups that participated in 60 days of strict, 6° head-down tilt (HDT) bed rest. Aim 1: posterior globe volume displacement compared with baseline (BDC-1) was measured at *HDT day 14* (*HDT14*), *HDT day 52* (*HDT52*), and at three days post-HDT (R+3). Aim 2: differences between the control and artificial gravity groups (intermittent artificial gravity, iAG; continuous artificial gravity, cAG) were assessed to test the effect of centrifugation as a mitigation technique.

The aims of this study were *1*) to quantify volume displacement of the posterior globe during and after 60 days of strict HDT bed rest and *2*) to assess the effectiveness of daily continuous or intermittent centrifugation as mitigation techniques (Fig. 2). To accomplish this, an automated MRI-based method was applied to segment the ocular globe and measure globe volume displacement (i.e., flattening).

## MATERIALS AND METHODS

### Study Design

In this randomized controlled study, subjects participated in a 60-day strict 6° HDT bed rest study with or without daily centrifugation as a means of artificial gravity (AG) at the :envihab facility of the German Aerospace Center (DLR) in Cologne, Germany. MRI scans were collected at four timepoints throughout the study: one day before bed rest (baseline data collection, BDC-1), on *days 14* (*HDT14*) and *52* (*HDT52*) of bed rest in the HDT position, and 3 days after bed rest (recovery, R+3) in the HDT position. The researchers involved in the data analysis (S.H.S., G.C.N., A.J.S., A.Q.F., and B.A.M.) were masked to participants’ subject groups and demographic information until after quantification of globe displacement was complete.

### Participants

Written informed consent was obtained from all participants in accordance with NASA IRB guidelines and approved by the ethics committee of the Northern Rhine Medical Association (Ärztekammer Nordrhein, Application No. 2018143) in Duesseldorf, Germany. Sixteen males and eight females successfully completed the study, two subjects were omitted from the final data set due to MRI artifacts before the researcher’s unblinding of study groups and demographics. Subjects were randomly assigned to one of the following groups: the intermittent artificial gravity group (iAG, *n* = 7), the continuous artificial gravity group (cAG, *n* = 8,), and the control group (ctrl, *n* = 7, Table 1).

**Table 1.** Demographics of study subjects by group

Group	Number of Subjects	Sex	BMI, kg/m^2^ (Means ± SD)	Age, yr (Means ± SD)
iAG	7	5 M, 2 F	24 ± 2	35 ± 11
cAG	8	5 M, 3 F	24 ± 2	32 ± 10
Control	7	6 M, 1 F	25 ± 3	33 ± 7
All Subjects	22	16 M, 6 F	24 ± 3	33 ± 9

Table showing the number of subjects, sex, BMI, and age for the intermittent artificial gravity (iAG), continuous artificial gravity (cAG), and control study groups.

### HDT Bed Rest

Participants remained in a tightly controlled environment for a total of 88 days, including ambulatory baseline data collection, 60 days of strict 6° HDT bed rest, and ambulatory recovery. During the 60 days of HDT, participants were required to keep at least one shoulder on the bed at all times and the use of pillows was not permitted to maintain a strict HDT posture and avoid confounding of results (a 5 cm head and neck support was allowed for side sleeping only) (10). All daily activities including eating and hygiene were performed in the HDT position. Diet was strictly controlled throughout the study with a daily energy intake of 1.3 times the resting metabolic rate and daily water intake of 50 mL/kg body mass. Twenty-four-hour monitoring by trained staff ensured compliance with all protocols.

### Artificial Gravity

**Figure 3.**
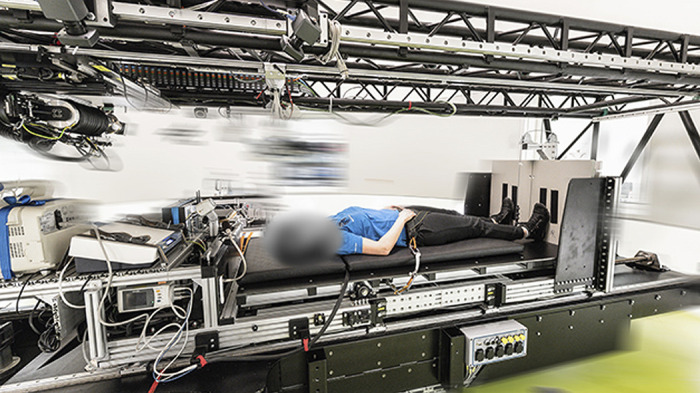
The short arm human centrifuge located at the :envihab research facility. The centrifuge spun at subject-specific rotational speed to generate an acceleration of 1 *g* at the center of mass and 2 *g* at the feet to simulate gravity for the intermittent artificial gravity (iAG) and continuous artificial gravity (cAG) study groups. Image source: DLR (German Aerospace Center), Cologne, Germany.

Participants in the artificial gravity (AG) groups completed daily sessions of centrifugation during the bed rest portion of the study. Gravity was simulated in the DLR short-arm centrifuge (Fig. 3) spun at a subject-specific rotational speed to achieve an acceleration of 1 g at the center of mass (∼2 g at the feet and 0.3 g at the eye) with rotational direction alternated daily. The cAG group completed daily continuous 30-min AG sessions whereas the iAG group completed six, 5-min bouts of centrifugation with 3-min breaks in between. Centrifugation occurred in the supine position (0° HDT), but subjects were positioned in 6° HDT immediately before and after each centrifuge run.

### MRI Acquisition and Reformatting

T2-weighted axial spin-echo fat-suppressed MRI sequences were collected using a 3 T system (Verio 3 T; vB19; Siemens Healthineers, Erlangen, Germany) with 0.78 mm in plane isotropic pixel size (field of view 100 × 100) and 0.78 mm slice thickness and spacing. Additional sequence parameters included a 170° flip angle, 750 ms repetition time, 112 ms echo time, and 123.21 Hz imaging frequency. Images were collected under 6° HDT.

### Point Cloud Generation and Alignment

**Figure 4.**
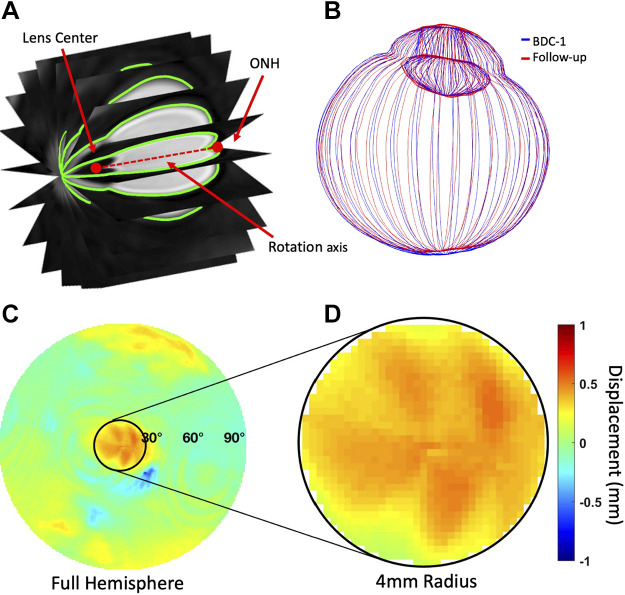
Methods for segmenting t2-weighted axial ocular MRI. *A*: images were radially resliced and segmented. *B*: baseline and follow-up globe point clouds were aligned using iterative closest point registration. *C*: displacement maps of the posterior globe were generated for each baseline and follow-up pair. *D*: posterior optic globe displacement was quantified within a 4 mm radius of the optic nerve head (ONH).

Each globe underwent an automated multistep three-dimensional (3-D) reconstruction and displacement mapping process in MATLAB (v.2019a, MathWorks Corp. Natick, MA) as previously described (7). In brief, each globe was radially resliced about a central rotational axis at one-degree increments in Osirix MD (v.8.0.1, Pixmeo, Geneva, Switzerland) and cubically upsampled by a factor of four (Fig. 4*A*). Segmentation of each slice was completed using an offset value of pixel intensity based on a global threshold to create a contour. Contours were transformed into 3-D coordinates on a common axis to create a point cloud of the globe. A box grid filter was applied (MATLAB computer vision toolbox, pcdownsample) to ensure uniform point distribution. An iterative closest point registration algorithm with 60 iterations (MATLAB computer vision toolbox, pcregistericp) was applied to align follow up point clouds to their respective baseline (BDC-1) point clouds (Fig. 4*B*). The spherical coordinates of each point on the posterior hemisphere of the globe surface were expressed in a two-dimensional (2-D) polar coordinate distance map. Circumferential and meridional angles of each point were transformed into polar angles and radial locations, respectively, and the distance between each of the points from the baseline point cloud’s centroid was represented by a color on the distance maps. The baseline distance map was subtracted from the follow-up distance map to generate a paired displacement map (Fig. 4*C*). Volume displacement was calculated for the region of the distance map within 4 mm of the optic nerve head to best represent the phenomenon of globe flattening (Fig. 4*D*). Positive values of displacement represent flattening of the posterior globe and negative values represent distension of the posterior globe. Changes in the total volume of the optic globe were not measured.

### Statistics

A linear mixed-effects model that accounts for repeated measurements from the same individual was developed:

$$y_{i}={\text{β}}_{0}+{\text{β}}_{1}x_{1i}+{\text{β}}_{2}x_{2i}+{\text{β}}_{3}x_{3i}+z_{0i}+z_{1i}x_{1i}+z_{2i}x_{2i}+z_{3i}x_{3i}+\epsilon_{i},$$

where *y_i_* is the measurement of a parameter of interest, β_0_ is the baseline (e.g., left eye of a male subject at BDC-1 and in the ctrl group), *x*_1_*_i_* is the treatment group, *x*_2_*_i_* is the timepoint, and *x*_3_*_i_* denotes the right eye of the *i*th subject, respectively. While coefficient β represents fixed effect sizes, coefficients *z*_1_, *z*_2_, and *z*_3_ represent the random effects of treatment group, timepoint, and eye, respectively.

The “*fitlme*” function in MATLAB (v.R2019a MathWorks Corp., Natick, MA) was used to estimate the coefficients and variances in this linear mixed-effects model and test the hypotheses. The model treats treatment group, timepoint, and eye as both fixed and random effects. Since the subjects were measured repeatedly, this mixed-effects model allows us to account for the variability in groups, timepoints, and eyes, across subjects. *P* values for globe volume displacement were calculated with this linear-mixed effects model.

## RESULTS

**Figure 5.**
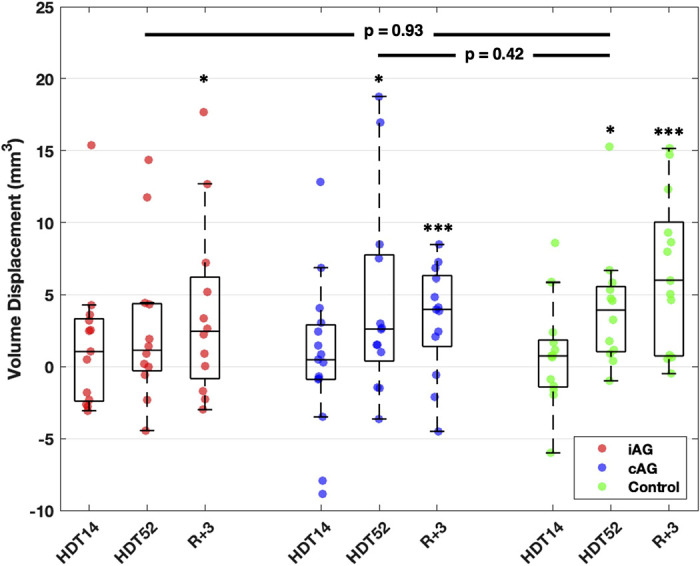
Plot showing volume displacement for the intermittent artificial gravity (iAG, red), continuous artificial gravity (cAG, green), and control groups (blue) at *day 14 head-down tilt* (*HDT14*), *day 52 head-down tilt* (*HDT52*), and three days post-HDT (R+3) with intra- and intergroup statistical significance as calculated by the linear mixed effects model (*P ≤ 0.05, ***P ≤ 0.005). On each box, the central mark indicates the median, and the bottom and top edges of the box indicate the 25th and 75th percentiles, respectively. The full range represents 1.5× the interquartile range. All displacements are referenced to pre-HDT baseline geometries.

Posterior globe volume displacement tended to increase with HDT bed rest duration and did not recover within three days post-bed rest for all three study groups (Fig. 5) (*P* < 0.001). For the iAG group, average volume displacement was 2.44 mm^3^ (*P* = 0.258) at *HDT14*, 4.74 mm^3^ (*P* = 0.075) at *HDT52*, and 5.47 mm^3^ (*P* = 0.027) at R+3 when compared with BDC-1 (Table 2). For the cAG group, average displacement was 0.85 mm^3^ (*P* = 0.67) at *HDT14*, 5.05 mm^3^ (*P* = 0.03) at *HDT52*, and 4.23 mm^3^ (*P* = 0.003) at R+3 when compared with BDC-1 (Table 2). For the control group, average displacement was 1.00 mm^3^ (*P* = 0.94) at *HDT14*, 3.51 mm^3^ (*P* = 0.04) at *HDT52*, and 6.86 mm^3^ (*P* = 0.001) at R+3 when compared with BDC-1 (Table 2).

**Table 2.** Volume displacement by group at each timepoint

Group	*HDT14* (Means ± SE, mm^3^)	*HDT52* (Means ± SE, mm^3^)	R+3 (Means ± SE, mm^3^)
iAG	2.44 ± 2.15	4.74 ± 2.66	5.47 ± 2.43*
cAG	0.85 ± 1.63	5.05 ± 2.39*	4.23 ± 1.37***
Control	1.00 ± 1.01	3.51 ± 1.11*	6.86 ± 1.47***

Table showing the mean, standard error (SE), and *P* value as calculated using the linear mixed-effects model of posterior globe volume displacement at head-down tilt (HDT) *day 14* (*HDT14*), HDT *day 52* (*HDT52*), and at three days post-HDT (R+3) for the iAG, cAG, and control study groups. cAG, continuous artificial gravity; HDT, head-down tilt; iAG, intermittent artificial gravity.

* *P* ≤ 0.05; ***P ≤ 0.005.

On average, differences between AG groups and the control group were not significant (*P* = 0.93 for iAG and *P* = 0.42 for cAG) (Fig. 5).

## DISCUSSION

Noninvasive automated techniques were applied to MRIs of HDT bed rest participants to quantify posterior optic globe volume displacement over time and assess the effectiveness of centrifugation as a mitigation technique. Small, inward displacements of the peripapillary choroidal-scleral interface were identified, with maximum displacement reported three days post-HDT. Neither 30 min of daily continuous nor intermittent centrifugation were effective in significantly reducing these displacements.

The methods used in this study segmented the optic globe near the choroidal-scleral interface; thus, the displacements measured are likely due to a combination of scleral flattening, and choroidal swelling. Another study of this cohort found that after 58 days of strict 6° HDT bed rest, total retinal thickness had increased by 27.6 μm and choroidal thickness remained largely unchanged (15). This suggests that the volume displacements measured here are primarily attributed to scleral flattening because they exceed that which could be explained by choroidal thickening alone. The same study also found no significant changes in refraction. This is because globe flattening that would cause changes in refractive error would need to extend to the macula, which is not observed in bed rest. Rather, here we report globe flattening at the optic nerve head. It is possible that when displacement is significant, as it is in astronauts, these changes could be detected at the macula.

Scleral flattening may be caused by a multitude of factors. Although a reduction or reversal of the TLPD has been hypothesized as a primary factor, other potential causes have yet to be identified. Six-degree HDT increases intracranial pressure (ICP) to a similar level of that seen in the supine posture (10, 20); however, this pressure is still elevated beyond what has been observed in the seated or standing positions (10). Chiquet et al. (21) measured IOP over seven days of 6° HDT and found that after an initial increase, IOP decreased from baseline by 0.96 mmHg after five days and 1.56 mmHg after seven days. In the current study, there were some increases in IOP reported, but they never exceeded the normal clinical range (15). The combination of chronically elevated ICP to supine levels creates a persistent reduction in the TLPD that may induce scleral flattening observed in the present study.

### Comparison to Astronauts

Significant globe volume displacement has been identified in astronauts after ∼6-mo spaceflight mission using identical methods (7). Average posterior globe volume displacement in the astronaut cohort was 9.88 mm^3^ ∼4 days after returning from long-duration spaceflight (167 ± 17 days), exceeding the displacements reported here (7). The most probable explanation for the difference is the significant choroidal thickening that has been identified in astronauts during spaceflight, but not in HDT subjects. The extended duration of spaceflight (∼6 mo) should also be considered. Other differences between spaceflight and this bed rest analog include the absence of all hydrostatic pressure vectors in spaceflight versus the alteration of the *Gz* (head to foot) vector only in bed rest studies, but not the *Gx* (chest to back) vector. Although this supports the use of strict, long-term HDT bed rest studies as a ground-based analog to spaceflight, it cannot be concluded that the globe displacement seen under HDT, and the displacement recorded after spaceflight result from the same underlying physiological stimulus. Although globe flattening was present in both cohorts, other spaceflight-associated findings such as decreased axial length and choroidal thickening were not identified in this HDT cohort (Table 3) (15).

**Table 3.** Differences between head-down tilt and spaceflight

Parameter	60 Days 6° HDT-BR	∼6 Months LDSF
Globe volume displacement (7)	**↑**	**↑↑**
Total retinal thickness (13)	**↑**	**↑**
Choroidal folds (3, 15)	**✓**	**✓**
Axial length (15)	**-**	**↓**
Choroidal thickening (8, 15)	**-**	**↑**
Intra-ocular pressure (15, 22)	**-**	**↑**

Table cataloging the similarities and differences in globe volume displacement, retinal, and choroidal thickening, choroidal folds, axial length changes, and intraocular pressure between strict head-down tilt bed rest (HDT-BR) and long-duration spaceflight (LDSF). A checkmark indicates the presence of the parameter, a dash indicates no change in the parameter, a down arrow indicates a decrease in the parameter, and an up arrow indicates an increase in the parameter with a double arrow signifying a much greater increase. ↑, increase; ↓, decrease; -, no change; ✓, present.

### Duration Interpretation

Posterior globe volume displacement tended to increase with HDT duration. At *HDT14*, none of the study groups showed statistically significant displacement, which suggests that the sclera is somewhat resistant to perineural anterior forces. However, by R+3, volume displacement increased significantly, suggesting that long-term alterations in the TLPD may result in remodeling of scleral tissue on a gradual and cumulative basis. This may explain why globe flattening in astronauts is not resolved within a year after returning from space (7). Average volume displacement was greatest 3 days post-HDT. This could be indicative of a lack of recovery or of the continued worsening of displacement that occurred during the 8 days of HDT-BR that took place following the HDT-52 timepoint.

### AG Interpretation

As differences in posterior globe volume displacement between the AG groups and the control group were not statistically significant, daily 30-min centrifugation sessions may not be of sufficient duration to significantly reduce globe flattening if mild but chronically elevated ICP is the cause. The typical adult will spend at least 16 h per day in the upright position, during which time ICP is well below supine levels (10). A 30-min duration is only ∼3% of the time a typical adult spends in the upright position; thus, a longer duration of daily AG is likely needed. Also, subjects only experienced 0.3 *g* at eye level, much less than experienced in the upright position on Earth.

### Limitations

There are several limitations that remain unaddressed in this study. This study uses a novel method for quantifying posterior globe displacement that needs to be validated for reliability and repeatability. However, previous applications of this method showed good agreement with ocular biometry measurements (7). Changes in the optic globe were only quantified within a 4 mm radius of the optic nerve head (ONH). This excludes any changes that could be occurring to the cornea, lens, muscle attachments, macula, or volume changes occurring to the entire globe. Although HDT bed rest was able to reproduce some of the effects of spaceflight, other spaceflight-associated findings were not reproduced (Table 3), suggesting that there are limitations to the use of HDT as a spaceflight analog. Another limitation is the low number of subjects in the study. Finally, the duration of centrifugation was a small fraction of the typical time spent by an adult in a lowered ICP state, with *G* forces acting in a gradient along the length of the body and a lower *G*-load at the level of the eye compared with the center of mass.

### Conclusions and Future Work

These findings suggest that strict, 60-day HDT bed rest is an appropriate ground-based analog for spaceflight and for studying SANS mitigation techniques. Daily 30-min sessions of neither continuous centrifugation nor intermittent centrifugation were sufficient to significantly reduce the degree of globe volume displacement. Future studies with longer centrifuge duration may result in a more significant reduction in globe flattening. Applying these methods in healthy subjects over time will help determine the repeatability of the methods and provide a measure of nominal changes in the posterior globe.

## GRANTS

This study was funded by NASA Grant No. 80NSSC20K0920, NASA Idaho Space Grant Consortium Grant No. NNX10AM75H, National Institute of Neurological Disorders and Stroke Grant No. 1R01NS111283-01.

## DISCLOSURES

B.A.M., S.H.S., and G.C.N. are full-time employees of Alcyone Therapeutics, Inc. B.A.M. has received grant support from Genentech, Minnetronix Neuro, Biogen, Voyager Therapeutics, and Alcyone Therapeutics. B.A.M. has been a scientific advisory board member for Alcyone Therapeutics and the Chiari and Syringomyelia Foundation and has served as a consultant to Roche, SwanBio Therapeutics, Cerebral Therapeutics, Behavior Imaging, Neurosyntek, Medtrad Biosystems, Minnetronix, Genentech, Praxis Medicines, Invicro, and CereVasc. None of the other authors has any conflicts of interest, financial or otherwise, to disclose.

## AUTHOR CONTRIBUTIONS

S.H.S., G.C.N., A.J.S., K.M.-G., S.S.L., B.R.M., and B.A.M. conceived and designed research; E.M.B. performed experiments; S.H.S., G.C.N., A.J.S., A.Q.F., and B.A.M. analyzed data; S.H.S., G.C.N., A.J.S., A.Q.F., D.S., K.M.-G., S.S.L., and B.A.M. interpreted results of experiments; S.H.S. prepared figures; S.H.S. drafted manuscript; S.H.S., G.C.N., A.J.S., A.Q.F., D.S., E.M.B., K.M.-G., S.S.L., B.R.M., and B.A.M. edited and revised manuscript; S.H.S., G.C.N., A.J.S., A.Q.F., D.S., E.M.B., K.M.-G., S.S.L., B.R.M., and B.A.M. approved final version of manuscript.
